# Regional differences, distributional dynamics and convergence of multidimensional food security levels in China

**DOI:** 10.1371/journal.pone.0309071

**Published:** 2024-08-16

**Authors:** Jing Cheng, Xiaobin Yu

**Affiliations:** Jiangsu University of Science and Technology, School of Humanity & Social Science, Zhenjiang, China; Sichuan Agricultural University, CHINA

## Abstract

Food security is one of the important issues in the current world development process. The article takes 31 provinces (districts and cities) in China as the research object and constructs a multidimensional food security level evaluation index system from four dimensions: quantitative security, nutritional security, ecological security, and capacity security. Using the entropy method, China’s food security index was calculated for the ten-year period from 2013 to 2022. Overall, China’s food security level showed an upward trend during the decade, with the provinces of Shandong, Heilongjiang, and Henan having the highest level of security. The distribution dynamics of food security and its spatiotemporal evolution in the seven regions were examined using the Dagum Gini coefficient and its decomposition, and the absolute and conditional convergence of food security in the different areas was verified. The results of the study show that the provinces within East China have the largest gap in food security levels between them, and there is absolute β-convergence. Looking at China as a whole, the development of its food security level is characterized by significant convergence, which means that provinces with a low level of food security will have a faster rate of growth than those with a high level of food security, resulting in a gradual narrowing of the gap in food security levels between provinces.

## Introduction

To promote Chinese-style modernization, it is necessary to persistently strengthen the foundation of agriculture and promote the comprehensive revitalization of the countryside. In Central Document No. 1 issued by China in 2024, one of the first tasks is ensuring national food security as the bottom line. The problem of feeding more than 1.4 billion people is China’s biggest livelihood and its biggest national condition. Only when a country is self-sufficient in food can it take the initiative in food security. Food security is an important foundation for economic development, social stability, and national security. However, the various social shocks that have occurred in recent years, including wars and conflicts, trade barriers, the frequent occurrence of extreme weather, and other overlapping factors, have all hurt China’s ability to ensure food security.

China’s food security faces a number of problems, including the difficulty of sustained production increases and the unbalanced distribution of security levels. On the economic front, Heilongjiang Province, which ranked first in per capita grain production in 2022, is nevertheless ranked 30th in the country in terms of per capita GDP. This mismatch between agricultural development and the economic situation still exists in many ways. In terms of climate, 22 national meteorological stations accumulated maximum temperatures that exceeded the extremes in the early summer of 2023. China’s average temperature has increased by 0.3 degrees per decade, significantly higher than the global average, which has led to significant changes in climate resources for Chinese agriculture.

In 1974, the Food and Agriculture Organization of the United Nations (FAO) first introduced the concept of "food security", which essentially refers to the assurance that any person can purchase enough food at any given time to sustain their survival and health. Internationally, the definition of food security tends to reflect the safety of dietary consumption, with the core meaning that anyone can satisfy his or her own needs for adequate and safe food [1]. In China, when evaluating the level of food security, it is more inclined to consider the food supply aspect, focusing on supply-side indicators such as food production and food self-sufficiency rate [2]. With the changes in socio-economic development and food consumption concepts, scholars’ definitions of the concepts and connotations of food security have also evolved with the times. For example, O. Charles Aworh emphasized that dietary diversity is the key to sustainable food security [3], Alisher Mirzabaev argued that food security and nutrition correlate with climate change [4], and Md. Ziaul Islam suggested that food security should take into account the phenomenon of food loss and waste [5]. In the context of the new development stage, scholars’ comprehensive examination of food security provides a theoretical basis for accurately grasping the basic connotation of national food security and promoting the implementation of the national food security strategy.

At present, the studies of scholars on multidimensional food security mainly focus on the construction of food security indicator system [6], food security assessment [7], influencing factors [8], and the guaranteed path of food security [9]. First, the construction and measurement research of the food security indicator system. The food security situation can be reflected by certain quantitative indicators, so the evaluation of food security first involves the construction of the indicator system. In the evaluation system, most of the studies start with the supply capacity and derive relevant indicators such as per capita grain production, unit area production, and grain production fluctuation coefficient [10]. Subsequent researchers expanded on this foundation, and the indicators of the evaluation system have gradually been made to cover a wide range of industries, including agriculture, manufacturing, transportation, and marine fisheries [11]. Romanus Osabohien constructed a green economy-based food insecurity evaluation mechanism [12]. Seungmin Lee identified the food insecurity situation of different countries by incorporating a new dynamic metric, the probability of food security (PFS), to identify different groups. This evaluation metric changes according to differences in demographic characteristics, income, and geographic location, resulting in an evaluation system with different patterns [13]. In the context of epidemic conflicts, David Laborde analyzes the risk of food security in terms of four dimensions: food availability, food access, nutrition, and stability [14].

Second is the assessment of food security. On the one hand, there is the use of assessment methods. Shishuzhu used remote sensing to calculate annual net primary productivity (NPP) to predict the annual production of food [15]. Cascade Tuholske used three indicators, namely, household food insecurity access scale (HFIA), household food insecurity access prevalence (HFIAP), and food consumption scores (FCS), to compare the level of food security at the household level [16]. Paramita Roy used maximum entropy (Maxent) and an analytic neural network (ANN) for food security and its vulnerability assessment [17]. On the other hand, there is the choice of assessment perspective. Xusiqing studied whether the delayed use of bioenergy crops threatens climate and food security from an ecological perspective [18]. Dibyajit Lahiri studied the use of bacteriocins in food security and preservation from a biological perspective [19]. Thalia M Sparling studied from a psychological perspective food security and nutrition about human mental health [20]. Most of the current evaluation studies on food security focus on quantitative analysis, and there is relatively little literature on spatial and temporal distribution and dynamic evolution.

Thirdly, research on factors affecting food security. The main factors that have been the subject of much research include arable land resources, international markets, climatic factors, economic factors, and political factors. When these factors negatively manifest themselves, they can lead to an overwhelming sense of "worry" about food security in society, which in turn can lead to a "worrying" status quo in the level of food security. Ni Guohua incorporated this "food security worry index" into the policy system to quantify the impact of "worry" on China’s food policy system from a multidimensional perspective [21]. P. Krishna Krishnamurthy R examines the relationship between early warning diagnosis of food insecurity and food security in terms of drought-related changes in food security [22]. Raul Gouvea assessed the role of technology in improving food security risks in the face of artificial intelligence changes, the digital age and concluded that technological innovation has a positive effect on food security worldwide [23].

Fourthly, the study of policies to safeguard food security. Different countries have different objectives, so the recommendations made by scholars are also varied. Khoa Vu argues that food relief policies should prioritize the few regions that are expected to be severely affected [24]. Liliane Abdalla calls for the government to formulate a multisectoral and non-discriminatory European integration policy [25]. In addition to the above studies, scholars have analyzed in numerous other directions, such as the impact of consumer shopping behavior of the population on food security [26], the extent to which unhealthy lifestyles of middle-aged and elderly people are associated with food insecurity [27], the impact of solar energy projects on food security [28], and so on.

In summary, the existing literature provides a rich theoretical basis and practical foundation for the study of food security, which helps to understand the connotation of food security and analyze the factors affecting it. However, the existing studies may also have the following shortcomings: first, most of the studies focus on quantitative food security, and there are fewer indicators in the evaluation system about the diversification of food supply types and supply channels. Since entering the new era, building a diversified food supply system is an inevitable choice to ensure food security, and accelerating the upgrading of food structure is an essential trend to meet people’s needs for a better life. Therefore, the food security evaluation system should be further enriched to make the food security measurement results more comprehensive. Second, research on food security is limited to agriculture alone, with fewer studies considering other sectors. Agriculture is the basis of human survival, but food security also involves the coordinated support of forestry, animal husbandry, fishery [29], industry, transportation, and other sectors. While food security in foreign countries focuses on food security, food security research in China has mainly focused on agricultural production and supply, possibly ignoring other areas involved in food security. Thirdly, the methodology of food security evaluation needs to be improved and refined. Food security strategies are constantly changing with the development of the times, and the analysis of the current situation of food security also needs to be updated and adjusted promptly. Existing literature is mostly based on theoretical discussions, lacking a systematic and comprehensive quantitative analysis of food security, and research on the spatial and temporal evolution of food security and convergence analysis is relatively small. Given the above shortcomings, this paper makes corresponding improvements. First, we add the indicators of total meat production, gross value of agriculture, forestry, animal husbandry and fishery, and road density to the evaluation system. The whole view and current situation of China’s food security are analyzed from four dimensions: quantitative security, nutritional security, ecological security, and capacity security. Second, the methodology of food security assessment is enriched. Based on China’s food security index from 2013–2022, the entropy method and comprehensive evaluation method are used to explore the level and distribution dynamics of China’s food security. Third, using the Dagum Gini coefficient and the convergence model, we analyze the regional differences in food security among China’s provinces and show the pattern of evolution of China’s multidimensional food security. This enables each region to design and select appropriate development goals based on its food security status quo and ultimately to achieve a unified whole of coordinated development.

## Methods

In this paper, we will take 31 provinces in China as the research object, construct a multidimensional food security evaluation system from four aspects of quantitative, nutritional, ecological, and capacity security, and use the entropy method to assign weights and calculate the food security index; then we will analyze the spatial and temporal differences in the distribution of China’s food security level by using Dagum Gini coefficient and its decomposition method. Finally, we will carry out a convergence analysis to explore the convergence characteristics of the food security level in different regions.

### Multidimensional food security indicator system

Based on the definition of the concept of food security in the existing literature, this paper starts from the multidimensional perspective of quantitative security, nutritional security, ecological security, and capacity security, keeping in mind the connotation of food security in the new era of the country, and finally constitutes an assessment system of food security level consisting of a total of 16 indicators in 4 dimensions. The multidimensional food security indicator system constructed as a result is shown in **Table 1**. The indicators in the system are categorized as positive and negative, with positive indicators showing a positive correlation with the level of food security and negative indicators showing a negative correlation.

**Table 1:** Multidimensional food security indicator system.

Dimension	Indicator	Indicator unit	Indicator characteristics	Weights
quantitative security	grain production	10000 tons	+	10.78%
	grain production per unit area	kg/hm2	+	2.86%
	per capita food production	kg per person	+	8.77%
	total output value of agriculture, forestry, animal husbandry and fishery	billion	+	7.89%
nutritional security	pesticide usage	ton	−	2.06%
	fertilizer usage	10000 tons	−	1.27%
	agricultural diesel usage	10000 tons	−	0.96%
	total meat production	10000 tons	+	8.47%
ecological security	area freed from waterlogging	1000 Ha	+	21.94%
	effective irrigated area	1000 Ha	+	9.87%
	disaster-affected area	1000 Ha	−	0.74%
	usage of agricultural plastic film	ton	−	1.38%
capacity security	road density	km/km^2^	+	5.67%
	agricultural production price index	-	−	1.53%
	total power of agricultural machinery	10000 KW	+	9.98%
	per capita disposable income	yuan	+	5.82%

In terms of quantitative security, food for the people is the most important thing, and guaranteeing the production quantity and production efficiency of food is the basis for measuring the level of food security. Ensuring food supply is the top priority of the country’s implementation of the rural revitalization strategy, and adequate food reserves can not only guarantee the supply of the food market but also maintain the basic stability of the market [30]. China adheres to the policy of basic food self-sufficiency based on domestic production, and continues to promote the structural reform of the agricultural supply side to improve the country’s overall food production capacity year by year and optimize the food supply structure. Referring to the research results of Cordell, D [31], we set the indicators of "grain production" and "grain production per unit area" to reflect China’s grain production capacity. At the same time, the indicators of "per capita food production" and "total output value of agriculture, forestry, animal husbandry, and fishery" are used to reflect the degree of food security and the supply capacity of the market in China. As supply-side indicators, the higher the value of these four indicators, the higher the level of food security.

In terms of nutritional security, the No. 1 document of the central government mentions that the construction of a diversified food supply system should be gradually promoted, so that people can establish the consumption concept of the big food view. Analyzing the level of food security from the perspective of a diversified food supply can fully reflect the degree of satisfaction of people’s needs for diversified food [32]. Referring to the research results of Will J. Brownlie [33], the three indicators of "pesticide usage," "fertilizer usage," and "agricultural diesel usage" are set to reflect the quality and safety of the food supply. "Total meat production" was used to reflect the diversification of food production and consumption by the population [34]. Of these, the three usage indicators indicate the level of quality of food in the production process, with higher values of the indicators being associated with lower levels of food security.

In terms of ecological security, it is not only necessary to reflect the level of utilization of food resources, but also to pay attention to the connotative characteristics of the sustainable development of food security [35]. At present, China is facing the challenges of the grim situation of arable land protection and the large impacts of natural disasters on agricultural production in guaranteeing food security, and it needs to pay continuous attention to the risk of gradually tightening resource and environmental constraints [36]. The indicators of "disaster-affected area" and "usage of agricultural plastic film" are used to evaluate the cultivation environment of Chinese grain and to measure the protection status and quality of arable land. Larger values indicate a poorer state of protection for arable land and a lower level of food security. At the same time, indicators of "area freed from waterlogging" and "effective irrigated area" are used to reflect the shift from quantitative to qualitative food production under the concept of sustainable development since the new phase.

In terms of capacity security, this dimension leans towards studying various infrastructure and policy measures that support food production and ensure people’s access to food. Due to the imbalanced distribution of food and barriers to food access, some regions, even with an abundant and stable food supply, cannot fully solve the problem of overall regional food security [37]. This leads to a paradox where production capacity exists but there is no access to food, and conversely, access exists but the capacity for productive output is not present. The two indicators "total power of agricultural machinery" and "road density" are used to examine the impact of basic support on the level of food security, and both showed a positive correlation with the level of food security. At the same time, the "agricultural production price index" and "per capita disposable income" are used to reflect the impact on the population’s ability to purchase food.

### Entropy scoring

When evaluating China’s multidimensional food security level, it is first necessary to objectively assign weights to the indicators and dimensions. The entropy value method can avoid the randomness problem of subjective bias and solve the problem of overlapping information between indicators. Therefore, this paper chooses the entropy value method to calculate the weights and scores of each indicator, and the calculation process is as follows:

(1) Standardize the data to make them comparable.

For the positive indicators:

$$r_{ij}=\frac{a_{ij}-min(a_{ij})}{max(a_{ij})-min(a_{ij})}$$ (1)

For the negative indicators:

$$r_{ij}=\frac{max(a_{ij})-a_{ij}}{max(a_{ij})-min(a_{ij})}$$ (2)

Where *r*_*ij*_ denotes the standardized value of indicator j in province i. *A*_*ij*_ denotes the original value of indicator j in province i.

(2) Calculate the weight of each evaluation indicator.


$$P_{ij}=\frac{r_{ij}}{\sum_{i=1}^{n}r_{ij}}$$ (3)

Where *P*_*ij*_ represents the ratio of the index value of province i to indicator j.

(3) Calculate the information entropy of each indicator.


$$E_{j}=‐1/lnn\sum_{i=1}^{n}p_{ij}\;\ln\;p_{ij}$$ (4)

(4) Calculate the utility value of information entropy for each indicator.


$$G_{j}=1-E_{j}$$ (5)

(5) Calculate the weights of indicators.


$$W_{j}=\frac{G_{j}}{\sum_{j=1}^{m}G_{j}}$$ (6)

(6) Calculate the combined food security score.

$$S_{i}=\sum_{j=1}^{m}W_{j}r_{ij}$$ (7)

where m denotes the number of indicators in the food security evaluation system, and *S*_*i*_ denotes the multidimensional food security index of province i.

### Dagum Gini coefficient

Compared with the traditional Gini coefficient, the Dagum Gini coefficient can accurately identify the sources of regional disparities and decompose the overall differences into three components: intra-regional, inter-regional, and hypervariable density. The Dagum Gini coefficient is a widely used tool in the study of regional differences and their causes. This method solves the problem of sample crossover that exists in traditional methods and analyzes the specific causes of regional differences [38]. In this paper, we use the Dagum Gini coefficient and its decomposition to analyze regional disparities in multidimensional food security in China with the following formula:

$$G=\frac{\sum_{j=1}^{k}\sum_{h=1}^{k}\sum_{i=1}^{n_{j}}\sum_{r=1}^{n_{h}}\left|y_{ij}-y_{hr}\right|}{2n^{2}\bar{y}}$$ (8)

where G denotes the overall Gini coefficient, k denotes the number of regions, n denotes the number of provinces, *y*_*ij*_(*y*_*hr*_) denotes the composite evaluation score of each province in region j(h), and $\bar{y}$ denotes the average of the multidimensional food security index for China as a whole.

According to the decomposition method of Dagum’s Gini coefficient, the overall Gini coefficient G is decomposed into intra-regional differences, inter-regional differences, and hypervariable density differences. This paper selects China’s officially released administrative region division method, which is divided into a total of seven regions: South China, North China, East China, Central China, Northwest China, Southwest China, and Northeast China. Intra-regional differences indicate the contribution of differences in food security indices among provinces within the seven regions, and inter-regional differences indicate the contribution of differences among the seven regions. Hypervariance density indicates the contribution of cross overlap between samples to the overall variation, which in this paper mainly refers to the increase in the overall variation caused by the presence of low-level provinces within regions with high levels of food security and the presence of high-level provinces within regions with low levels of food security.

### Convergence analysis

This paper focuses on σ-convergence and β-convergence analyses to explore whether regional differences in China’s multidimensional food security levels tend to converge or diverge, and to analyze whether regions with low levels of food security can catch up with regions with high levels of security at a faster pace.

σ-convergence refers to the declining trend of the multidimensional food security level deviation between different regions over time, and by calculating the coefficient of variation of the degree of food security, it is possible to determine whether or not there is convergence in the level of food security. If the coefficient of variation decreases from year to year, it means that the difference in food security level between different regions is gradually decreasing, i.e., there is σ-convergence at this time. In this study, the coefficient of variation is utilized to portray the convergence of σ. The specific formula is as follows:

σ=∑i=1n(Di,t−Di,t¯)2nDi,t¯ (9)

Where i denotes different provinces and t denotes the year, the multidimensional food security index of province i in year t is denoted by *D*_*i*,*t*_.

The theory of β-convergence analyzes whether, over time, regions with low levels of multidimensional food security can catch up with regions with high levels of security at a faster rate, thus gradually reducing the differences between regions. Eventually, both regions with high and low levels of food security will reach converging levels of growth [39]. This convergence phenomenon can be further categorized into two forms: absolute β-convergence and conditional β-convergence. Absolute β-convergence refers to the fact that the multidimensional food security levels of all regions will eventually converge to the same steady-state level, regardless of the initial gap in food security levels. Conditional β-convergence means that controlling for the factors affecting the level of multidimensional food security, the level of food security in each region will show a tendency to converge. This means that despite the differences in multidimensional food security levels in different regions, all will converge to a similar steady-state level.

Using *D*_*i*,*t*_ to denote the degree of multidimensional food security in region i in year t, *D*_*i*,*t*+1_ to denote the degree of multidimensional food security in year t+1, and *μ*_*i*_, *ν*_*t*_, and *ε*_*it*_ to denote the spatial fixed effects, the temporal fixed effects, and the random perturbation term, the computational models for absolute β-convergence and conditional β-convergence are as follows:

$$\ln(\frac{D_{i,t+1}}{D_{i,t}})=\alpha +\beta \;\ln\;D_{i,t}+\mu_{i}+\nu_{t}+\varepsilon_{it}$$ (10)

$$\ln(\frac{D_{i,t+1}}{D_{i,t}})=\alpha +\beta \;\ln\;D_{i,t}+\lambda \sum_{j=1}^{n}{Control}_{i,t}+\mu_{i}+\nu_{t}+\varepsilon_{it}$$ (11)

*α* is the constant term, *β* is the convergence coefficient, if *β* is significantly less than 0, it means that there is a convergence feature of multidimensional food security level, and vice versa, there is a dispersion feature, and the speed of convergence can be calculated by −ln(1+*β*)/*T*. *λ* denotes the coefficients of control variables, and *Control*_*i*,*t*_ denotes the control variables affecting the degree of multidimensional food security, which mainly include the area of arable land, the per capita disposable income of the residents, and grain production.

### Data sources

According to the data availability, this study selects the balanced panel data of 31 provinces (autonomous regions and municipalities) in China for 2013–2022 from the China Statistical Yearbook, the China Agricultural Yearbook, the China Water Resources Statistical Yearbook, as well as some local and municipal statistical yearbooks, and the missing data of individual years are made up by the interpolation method.

### Empirical analysis

#### Results of the food security index measure

According to the entropy method, by substituting the data into the formula, we can get the weight distribution of different dimensions in the evaluation system, and the results are shown in **Table 1**. Among these, quantitative security occupies 30.3% of the weight, nutritional security is 12.8%, ecological security accounts for 33.9%, and capacity security is 23.0%. It is worth noting that quantitative and ecological security have relatively high weights, accounting for more than 60% of the total, which fully emphasizes the centrality of quantitative security in guaranteeing food security. At the same time, with China’s consistent implementation of the sustainable development strategy, the importance of ecological security has also become more and more prominent, reflecting China’s deep understanding of and high concern for both quantitative and ecological food security. This weighting not only objectively reflects China’s long-term investment in and attention to multiple dimensions of food security, but also emphasizes the urgency and importance of strengthening the environmental protection of agricultural resources and promoting the green transformation of agricultural development. At the level of each indicator, the two indicators of "grain production" and "area freed from waterlogged" are particularly critical and account for 10.78% and 21.94%, respectively. This reflects the fact that food production and natural disaster prevention play a crucial role in guaranteeing food security. Governments at all levels are actively implementing the assessment of the responsibility system for arable land protection and food security, setting clear production targets, striving for a good grain harvest, promoting the stable development of animal husbandry, and pushing forward the development of modern fisheries. The area freed from waterlogging reflects the area where flood-removing standards are required to reach once every three years and above through the construction of flood-control projects or the deployment of water conservancy facilities such as flood-discharging machinery, which exempts originally flood-prone arable land from the threat of inundation and flooding. This refined indicator not only helps to accurately assess the risk-resistant capacity of agricultural production, but also reflects the country’s comprehensive consideration and precise policy in guaranteeing food security.

Based on the entropy value method, the food security index of each province is calculated using the comprehensive evaluation method, and the results are reported in **Table 2**. As a whole, according to the total score summarized over ten years, the top five provinces are Shandong, Heilongjiang, Henan, Jiangsu, and Anhui, which is consistent with the division of China’s main grain-producing areas and fully verifies the reliability and accuracy of the evaluation system. As an important part of the North China Plain, Shandong’s fertile arable land resources and favorable climate conditions provide a unique advantage for grain production. Heilongjiang is one of the largest grain-producing provinces in China, and its vast land area and rich agricultural resources lay a solid foundation for high and stable grain production. Other provinces, such as Henan, Jiangsu, and Anhui, also play a leading role in modeling food security, which together form a solid barrier for China’s multidimensional food security. The excellent performance of these provinces in terms of food security is not only due to their natural conditions and production capacity, but also to their efforts in terms of ecological and nutritional security.

**Table 2:** Multidimensional food security index by province.

	2013	2014	2015	2016	2017	2018	2019	2020	2021	2022
Chongqing	0.1932	0.1989	0.2059	0.2099	0.2110	0.2167	0.2194	0.2250	0.2405	0.2419
Zhejiang	0.2216	0.2283	0.2276	0.2412	0.2318	0.2352	0.2343	0.2391	0.2503	0.2501
Yunnan	0.2227	0.2323	0.2362	0.2424	0.2526	0.2563	0.2567	0.2664	0.2924	0.3024
Xinjiang	0.2299	0.2350	0.2478	0.2439	0.2519	0.2534	0.2634	0.2656	0.2801	0.3184
Tibet	0.1018	0.1058	0.1241	0.1220	0.1128	0.1118	0.1082	0.1088	0.1162	0.1210
Tianjin	0.1657	0.1688	0.1737	0.1769	0.1845	0.1813	0.1855	0.1856	0.1904	0.1971
Sichuan	0.3169	0.3270	0.3350	0.3402	0.3449	0.3513	0.3431	0.3623	0.3840	0.3866
Shanghai	0.1774	0.1841	0.1875	0.1854	0.2003	0.2054	0.2081	0.2074	0.2143	0.2148
Shaanxi	0.1804	0.1864	0.1930	0.1906	0.1930	0.1978	0.1976	0.2034	0.2143	0.2156
Shanxi	0.1832	0.1898	0.1915	0.1852	0.1854	0.1858	0.1830	0.1946	0.2032	0.2099
Shandong	0.6036	0.6211	0.6309	0.6054	0.6395	0.6481	0.6417	0.6592	0.6836	0.6978
Qinghai	0.0886	0.0952	0.0980	0.0960	0.0987	0.1017	0.1007	0.0970	0.1064	0.1095
Ningxia	0.1305	0.1383	0.1391	0.1402	0.1421	0.1451	0.1467	0.1463	0.1489	0.1562
Inner Mongolia	0.2748	0.2781	0.2832	0.2769	0.2977	0.3137	0.3225	0.3269	0.3422	0.3732
Liaoning	0.3002	0.2856	0.3018	0.3019	0.3087	0.3014	0.3161	0.3166	0.3382	0.3416
Jiangxi	0.2474	0.2566	0.2595	0.2612	0.2705	0.2743	0.2775	0.2837	0.3028	0.3084
Jiangsu	0.4807	0.4959	0.5077	0.5132	0.5669	0.5845	0.5913	0.5984	0.6135	0.5920
Jilin	0.3251	0.3255	0.3356	0.3396	0.3565	0.3337	0.3476	0.3516	0.3741	0.3855
Hunan	0.3339	0.3488	0.3525	0.3552	0.3616	0.3677	0.3608	0.3699	0.4019	0.3973
Hubei	0.3477	0.3625	0.3747	0.3693	0.3924	0.3998	0.3948	0.3902	0.4232	0.4312
Heilongjiang	0.5365	0.5514	0.5622	0.5571	0.6079	0.6089	0.6158	0.6332	0.6572	0.6644
Henan	0.5482	0.5671	0.5820	0.5664	0.5816	0.5920	0.5849	0.6022	0.6215	0.6405
Hebei	0.4441	0.4520	0.4557	0.4344	0.4480	0.4494	0.4540	0.4631	0.4738	0.4809
Hainan	0.1247	0.1261	0.1321	0.1399	0.1380	0.1487	0.1488	0.1504	0.1590	0.1623
Guizhou	0.1736	0.1902	0.1994	0.1983	0.2131	0.2152	0.2113	0.2136	0.2376	0.2400
Guangxi	0.2331	0.2384	0.2421	0.2427	0.2466	0.2531	0.2463	0.2524	0.2769	0.2779
Guangdong	0.2630	0.2703	0.2746	0.2812	0.2832	0.2891	0.2954	0.3035	0.3195	0.3231
Gansu	0.1445	0.1493	0.1545	0.1507	0.1546	0.1605	0.1637	0.1718	0.1820	0.1890
Fujian	0.1870	0.1930	0.1953	0.2108	0.2054	0.2089	0.2129	0.2206	0.2284	0.2314
Beijing	0.1609	0.1606	0.1600	0.1639	0.1682	0.1682	0.1691	0.1700	0.1801	0.1789
Anhui	0.4353	0.4502	0.4622	0.4635	0.4849	0.4912	0.4965	0.5057	0.5289	0.5356

In terms of provinces, the food security indexes of all provinces generally show an upward trend, which reflects the steady development of food production and the progress made in food security in each province. However, in specific years, such as 2016 and 2019, most of the provinces showed a decrease in the food security index. The main reasons for this may lie in the following: in 2016, meteorological conditions were poorer than in previous years, disasters were more frequent, and pests and diseases occurred more frequently in some areas than in other years. The frequent occurrence of natural disasters brought severe challenges to food production, especially dominated by large grain provinces, leading to a decline in food production in some regions. In 2019, the global agricultural industry chain successively encountered the impact of multiple negative impacts such as drought and conflict, and the uncertainty in the food market greatly increased, which brought great economic pressure and adverse impacts on China’s domestic food security. It is thus evident that climate change and natural disasters remain key causes of food insecurity.

**Fig 1:**
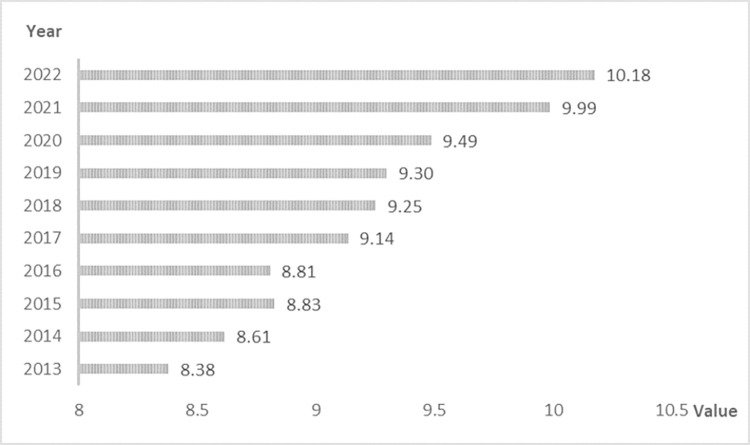
China’s comprehensive multidimensional food security score.

**Fig 1** illustrates the changes in China’s overall multidimensional food security index over 10 years. In terms of the process of change, China’s food security level has generally shown an upward trend, which is reflected not only in the steady increase in food production but also in several aspects, including the improvement of the ecological environment, the enhancement of access capacity, and the stable supply of the food market. The combined effect of these factors has made China’s multidimensional food security level overall show positive and upward development. A decline occurred in 2016, a year in which poor climatic conditions and frequent natural disasters reduced grain production in most provinces, leading to a decline in China’s overall food security level. The fastest growth in the food security index was recorded in the period from 2020 to 2021, with a growth rate of 5.28%. This phase was affected by epidemics, droughts, and conflicts, among others, and the international food market was highly volatile. However, China’s ample domestic reserves and grain stocks and well-developed emergency processing capacity ensured that the country’s grain market was supplied in sufficient quantities and at a stable price, providing support for addressing various risk challenges.

### Spatial and temporal differences and distributional dynamics

#### Dagum Gini coefficient

The Dagum Gini coefficient is a measure to study imbalances. First: the overall Dagum Gini coefficient = within group + among group + hypervariable density Gini coefficient; second: the contribution rate is the share of within group, among group, and hypervariable density Gini coefficients. Based on the food security index calculated above, the measurement and decomposition of the Dagum Gini coefficient is carried out, and the coefficient value and contribution rate of each component can be finally obtained, as shown in **Table 3**.

**Table 3:** Gini coefficient and contribution rate.

year	Gini coefficient				Contribution rate		
	overall	within	among	hypervariable	within	among	hypervariable
2013	0.274	0.032	0.181	0.061	11.690%	66.120%	22.191%
2014	0.272	0.032	0.179	0.062	11.752%	65.573%	22.675%
2015	0.270	0.032	0.177	0.061	11.748%	65.546%	22.706%
2016	0.264	0.03	0.175	0.058	11.557%	66.420%	22.023%
2017	0.274	0.032	0.181	0.061	11.587%	66.232%	22.182%
2018	0.273	0.032	0.178	0.063	11.717%	65.172%	23.111%
2019	0.272	0.032	0.178	0.063	11.709%	65.294%	22.998%
2020	0.273	0.032	0.176	0.065	11.846%	64.553%	23.602%
2021	0.270	0.032	0.174	0.064	11.733%	64.644%	23.623%
2022	0.267	0.032	0.169	0.066	11.961%	63.378%	24.661%

**Table 3** demonstrates the overall Gini coefficient and the contribution of each component to China’s multidimensional food security. The overall Gini coefficient shows an overall downward trend, indicating a gradual narrowing of the gap between the levels of multidimensional food security in China’s provinces. Two coefficient rebounds occurred during the study period, respectively, the Gini coefficient increased from 0.264 to 0.274 in 2017 and from 0.272 to 0.273 in 2020. In 2017, provinces actively promoted the structural reform of the agricultural supply side, adjusted the structure of grain cultivation, and the low-yielding areas gradually withdrew from wheat production. The frequent occurrence of persistent cloudy and rainy days in some provinces in the middle and lower reaches of the Yangtze River led to a reduction of the local grain acreage in winter. In addition, the focus of agricultural reform policies varies by province, for example, the implementation of cotton target price reform in Xinjiang has increased farmers’ incentives to plant cotton. In 2020, provinces were hit differently by the impact of the new Crown pneumonia epidemic. Provinces such as Hubei, Guangdong, and Henan were less efficient in resuming work and resuming production under severe epidemic control measures. Provinces with high net population inflows and high dependence on foreign trade were significantly more affected. In contrast, the impact on the western region was smaller. These factors have hampered the performance of food production in the provinces and exacerbated regional disparities in food security levels.

When analyzed in terms of dynamic changes, the contribution of within-group and between-group variance and hypervariance density to the overall variance varied inconsistently. The contribution of intra-group variation is the smallest. And the degree of fluctuation is also relatively smooth. In terms of physical geographic characteristics, provinces within the same region have similar endowments of elements such as topography, climate, soil, resources, and vegetation. In terms of human geographic features, the distribution of urban economic development clusters reflects the characteristics of close economic ties and convenient transportation networks, and there is agglomeration in agricultural development, industry, and transportation. The contribution rate of inter-group differences is the highest, accounting for more than half of the total, which indicates that China’s food security level has a more serious geographical imbalance and uneven distribution and that the gap in food security level between regions is still a problem that needs to be paid attention to in the long term. China is a vast country with different terrain heights and landscape types and thus has a wide variety of farming systems and cropping structures. For example, Beijing, Henan, and Qinghai are located in three geographic regions. Beijing’s agricultural volume is relatively small, and its supply is not enough to meet food consumption demand, but its economic strength and green development of agriculture guarantee Beijing’s food security. Henan Province, as a large agricultural and livestock province with a wide variety of food crops and huge yields, has a huge advantage in food production, but the accessibility of the province’s residents is slightly insufficient. Qinghai Province, with its high terrain and cold climate, mainly focuses on animal husbandry, with less overall grain production and a lower level of local economic development. Each province has to adapt to local conditions and explore an agricultural structure that suits the province according to its natural conditions and economic level.

**Fig 2:**
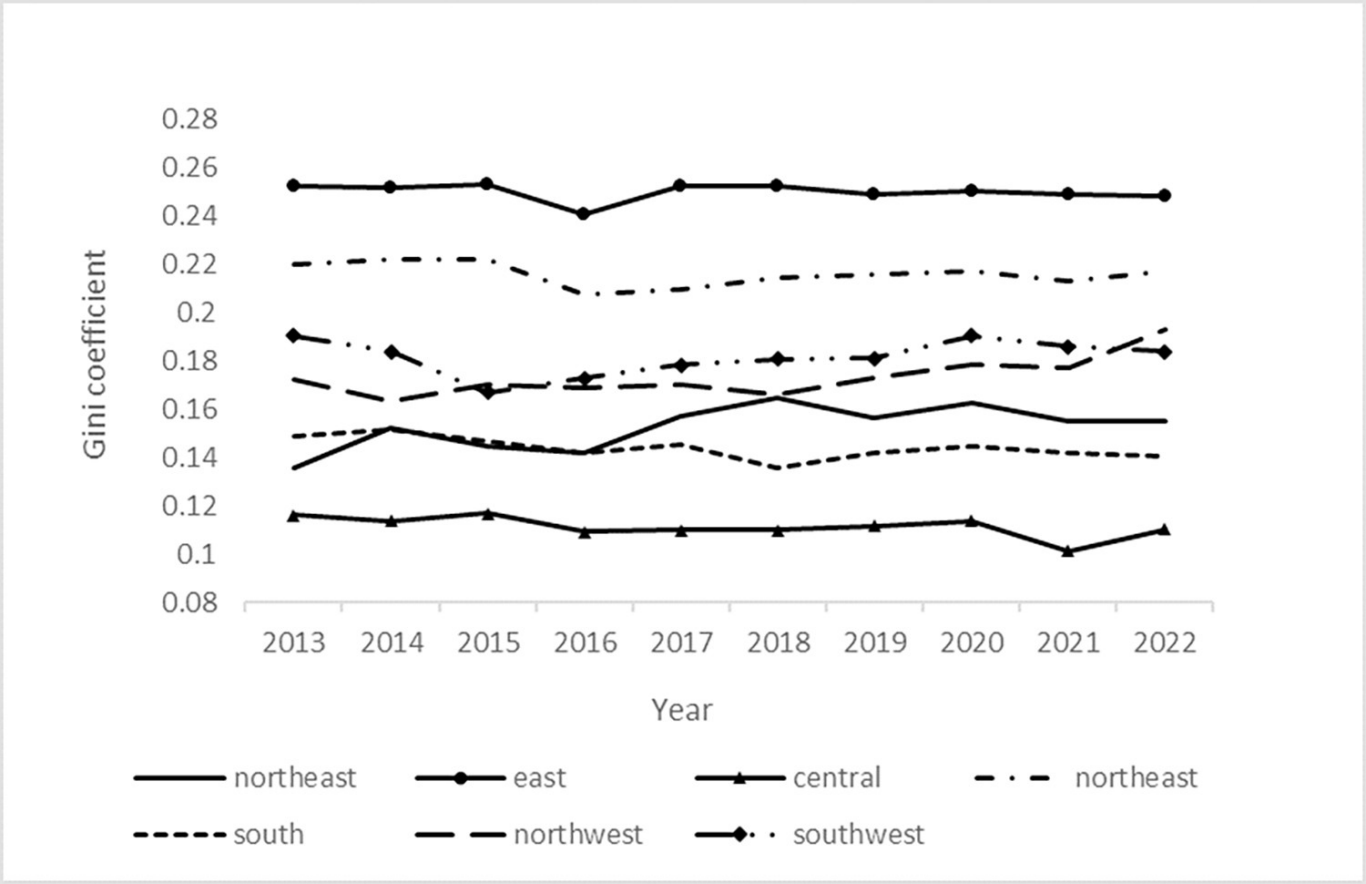
Within the Gini coefficient.

As can be seen in **Fig 2**, the Gini coefficient is the highest within the East China group, with the coefficient fluctuating around 0.25 in all years except for 2016, when it dropped to 0.24, indicating that the gap in food security levels is the largest within the region. Within East China, Jiangsu and Anhui are both large grain provinces with vast plains and relatively abundant arable land resources. Shanghai and Zhejiang, compared to the shortfall, these two provinces are mainly to develop high-tech, mainly industrial, in the acquisition of food is more dependent on the outside world, thus leading to a large gap in food security levels between the provinces within the East China region. Central China has the smallest intra-group Gini coefficient, and the three provinces in the region have a similar structure of agricultural development and all have a high level of food security.

### Convergence analysis

After analyzing the distribution dynamics of multidimensional food security and the decomposition of differences, to further explore the evolutionary trend of the differences in the levels of multidimensional food security in China, this paper uses σ-convergence and β-convergence models to analyze the convergence characteristics of the differences between different regions.

### σ-convergence test

**Table 4** demonstrates the evolutionary trend of the multidimensional food security coefficient of variation in China. China’s overall coefficient of variation shows a decreasing trend, indicating that the differences in food security levels between different provinces in China are narrowing, showing a significant σ-convergence feature. Specifically, it decreases from 0.503 in 2013 to 0.483 in 2016, and then from 0.504 in 2017 to 0.485 in 2022. The value of the coefficient of variation is the highest in East China, indicating that the food security gap between provinces within East China is the largest, which is consistent with the results of the Gini coefficient analysis above. After 2020, the coefficients of variation in the Northeast, East China, South China, and Southwest China regions all show a downward trend, suggesting that the differences in food security levels within the regions are narrowing and that synergistic effects between regions are increasing. Northwest China, North China and Central China have larger changes and do not show significant σ-convergence characteristics, i.e., the food security gap between regions has not continued to weaken, and spatial disparity imbalance still exists.

**Table 4:** Coefficient of variation.

	2013	2014	2015	2016	2017	2018	2019	2020	2021	2022
northeast	0.2737	0.3020	0.2891	0.2815	0.3092	0.3326	0.3153	0.3266	0.3124	0.3082
north	0.4371	0.4376	0.4358	0.4107	0.4135	0.4178	0.4211	0.4197	0.4111	0.4130
east	0.4645	0.4627	0.4665	0.4408	0.4659	0.4665	0.4617	0.4627	0.4577	0.4554
south	0.2870	0.2922	0.2818	0.2694	0.2771	0.2585	0.2647	0.2702	0.2696	0.2662
central	0.2389	0.2342	0.2368	0.2240	0.2184	0.2185	0.2206	0.2313	0.2051	0.2196
northwest	0.3082	0.2928	0.3052	0.3037	0.3069	0.2979	0.3115	0.3189	0.3164	0.3538
southwest	0.3475	0.3390	0.3102	0.3184	0.3300	0.3350	0.3331	0.3487	0.3423	0.3367
overall	0.5030	0.5019	0.4972	0.4834	0.5041	0.5031	0.5008	0.5035	0.4924	0.4857

### The absolute β-convergence

**Table 5** demonstrates the status of absolute β-convergence for multidimensional food security levels in China. As can be seen from the table, only China as a whole and East China and Southwest China have significantly negative absolute β-convergence coefficients at least at the 5% level, indicating the existence of absolute β-convergence in these regions. This means that regions with low levels of multidimensional food security will have faster development speeds and can catch up with regions with high levels of multidimensional food security by utilizing the "catching-up effect", without considering other influencing factors and other variables. Finally, the food security level of each region will converge to the same steady state level over time. In contrast, there is no absolute β-convergence in North China, Northeast China, South China, Central China and Northwest China, indicating that the gap between food security levels in these regions is still obvious and that it will be difficult to converge to the same level of security in a short period.

**Table 5:** The absolute β-convergence.

variable	overall	north	northeast	south	east	central	northwest	southwest
convergence factor	-0.083*** (0.032)	0.072 (0.082)	-0.089 (0.128)	-0.041 (0.082)	-0.129** (0.054)	-0.157 (0.118)	0.026 (0.088)	-0.173** (0.086)
constant term	-0.090** (0.043)	0.121 (0.118)	-0.064 (0.119)	-0.039 (0.127)	-0.122** (0.060)	-0.112 (0.099)	0.074 (0.161)	-0.242* (0.133)
area fixed effect	yes	yes	yes	yes	yes	yes	yes	yes
time fixed effect	yes	yes	yes	yes	yes	yes	yes	yes
R^2^	0.026	0.019	0.021	0.011	0.094	0.072	0.002	0.095
F	0.65	1.49	0.32	0.14	1.18	0.83	0.34	1.34

Note: *, **, *** indicate significance at the 10%, 5%, and 1% levels, respectively, with standard errors in parentheses.

### The conditional β-convergence

The formula for conditional convergence is used to calculate the conditional β-convergence coefficient of multidimensional food security from 2013 to 2022, and the specific results are shown in **Table 6**. Based on the regression results, it can be seen that conditional β-convergence exists in China as a whole, in the Northeast, in South China, in Central China, in Northwest China, and in Southwest China, i.e., with time, the multidimensional food security of each province converges to its respective steady state level. In North China and East China, the convergence coefficients are negative but not significant, indicating that there is no conditional β-convergence in these two regions.

**Table 6:** The conditional β-convergence.

variable	overall	north	northeast	south	east	central	northwest	southwest
convergence factor	-0.23*** (0.05)	-0.02 (0.14)	-0.50*** (0.17)	-0.43* (0.22)	-0.17 (0.10)	-0.75*** (0.24)	-0.41** (0.20)	-0.40** (0.14)
grain production per unit area	-0.00***	-0.00	-0.00***	-0.00	-0.00***	-0.00	0.00*	0.00
per capita disposable income	0.00***	0.00	0.00***	0.00**	0.00**	0.00***	0.00	0.00**
constant term	-0.26** (0.10)	-0.02 (0.25)	-0.40 (0.24)	-0.69 (0.44)	-0.04 (0.16)	-0.61** (0.26)	-1.05** (0.45)	-0.81** (0.38)
R^2^	0.18	0.08	0.71	0.30	0.38	0.51	0.17	0.26
F	1.84***	0.91	14.66***	1.81	2.93**	5.27**	1.63	1.83

Note: *, **, *** indicate significance at the 10%, 5%, and 1% levels, respectively, with standard errors in parentheses.

## Discussion

China’s land area is widely distributed, and there are obvious gaps in the level of development of the food industry in various regions. There are still more challenges on the way to improve the food security level, and the research on food security is a long-term task. This study analyzes the spatio-temporal differences in regional food security in China using the Dagum Gini coefficient and its decomposition and convergence analysis. It is found that the spatio-temporal characteristics of food security in China are closely related to the spatial distribution of food production and per capita income. China’s overall food security level is gradually improving, and the development gap between provinces is gradually narrowing. Compared with previous studies, the selection of food security evaluation indicators in this paper is not limited to the traditional food supply side, but is comprehensively considered from multiple dimensions. The new food security measurement system not only provides a theoretical reference for food security research, but also provides decision-making reference for the improvement of food security level.

Food security is typically a cross-disciplinary issue, and many aspects of research have emerged [40]. For example, the combination with various factors such as marine fisheries, climate change, bioenergy and finance have all increased the comprehensiveness of research on the level of food security. This study focuses on quantitative security, nutritional security, ecological security and capacity security, which directly reflect the level of food security in China. Therefore, this study’s discussion of food security provides a new perspective for other scholars. By focusing on multidimensional factors, the results of the study can reflect the state of food security more comprehensively and accurately.

However, this study still has some limitations. Due to the small time span of the study and the level of analysis, it still needs to be improved. Food security is an important guarantee for world peace and development, and is related to the sustainable development and future destiny of mankind [41]. Future research can analyze the situation of food security from multiple disciplines and perspectives. The process of food production is a complex system engineering involving multiple disciplines and fields such as biology, geography and agronomy. By mastering this composite disciplinary background, it is conducive to broadening the channels of food security and providing effective support for food security strategies. In addition, it is also possible to focus on characteristics such as distribution dynamics and spatial and temporal differences according to the division of different administrative regions, with a view to narrowing the development gap in food security levels between regions.

## Conclusion

This paper measures China’s food security in terms of quantitative, nutritional, ecological and capacity security. Currently, most of China’s food security studies at the inter-regional level focus on the level of food security and the path to security. By studying the distribution characteristics and dynamic convergence of food security at the regional level, the main conclusions are obtained as follows. China’s food security level shows an upward trend from 2013 to 2022, with Shandong and Heilongjiang provinces having the highest level of security. The multidimensional food security level gap among provinces is gradually narrowing, with the largest share of the gap caused by inter-region, both exceeding 60%. Finally, there is absolute β-convergence and conditional β-convergence in China as a whole. Grain production per unit area and per capita income play an important role in promoting food security, while there is no convergence in the differences between regions, controlling for the variable "affected area".

Enhancing the level of food security and narrowing the gap in the level of security between regions requires a number of policies to be implemented and safeguarded. The following policy recommendations of this study are conducive to providing assistance in upgrading the level of food security. First of all, the task of focus still depends on the development of large grain-producing provinces and the advantages of the main grain-producing regions. Ensure that arable land is in good condition in terms of both quantity and quality to increase grain production from the source of supply. Secondly, it is necessary to optimize the structure of agricultural cultivation in the western region, raise the income of residents, improve transportation facilities and increase the efficiency of grain circulation. The western region has obvious deficiencies in both agricultural and economic development, and the government should increase financial support to promote the cross-regional flow of advantageous resources. Finally, the protection of the ecological environment should be strengthened, and the use of pesticides and fertilizers in the planting process should be reduced, so as to enhance the planning capacity of the arable ecosystem.

## Supporting information

S1 Data (XLSX)
